# Microarray analysis of the effects of Acthar Gel versus methylprednisolone in a model of focal segmental glomerulosclerosis in female rats

**DOI:** 10.14814/phy2.70321

**Published:** 2025-04-13

**Authors:** Kyle Hayes, Dale Wright

**Affiliations:** ^1^ Mallinckrodt Pharmaceuticals Bridgewater New Jersey USA

**Keywords:** Acthar Gel, fibrosis, focal segmental glomerulosclerosis, gene expression, inflammation

## Abstract

Acthar® Gel (repository corticotropin injection) is an alternative treatment for patients with focal segmental glomerulosclerosis (FSGS) who cannot tolerate or do not adequately respond to glucocorticoids or calcineurin inhibitors. We compared the effects of Acthar versus methylprednisolone (MP) on gene expression in the kidney cortex in a rat model of FSGS induced by puromycin. Female Sprague–Dawley rats (6–8 weeks old) were treated for 8 weeks with Acthar 60 IU/kg (*n* = 5), MP 2 mg/kg (*n* = 5), or control (*n* = 4). On Day 56, animals were sacrificed, and RNA samples of kidney cortex tissue were analyzed using microarrays. Compared with control, Acthar significantly decreased the expression of more genes related to inflammation, immune function, and fibrosis than MP. A subset of these genes exhibited significantly larger fold changes in expression after treatment with Acthar versus MP, including *C1qb* and *C1qc* (complement cascade), *Ccr2* and *Tcrb* (immune function), and *Mfap4* and *Vim* (fibrosis). These results suggest that Acthar acts as an immunomodulator with a distinct mechanism of action from that of MP. Their differential alteration of gene expression in the kidney cortex suggests that Acthar may be more effective than MP in reducing inflammation and fibrosis in FSGS, which could slow disease progression.

## Graphical abstract

Effect of Acthar^®^ Gel versus methylprednisolone on gene expression in a female rat model of focal segmental glomerulosclerosis.

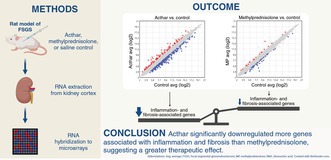

## 1 INTRODUCTION

Focal segmental glomerulosclerosis (FSGS) is the most common cause of nephrotic syndrome in adults in the US (Fogo, 2015). The pathogenesis of FSGS involves podocyte injury and depletion, which create gaps in the glomerular filtration barrier and lead to proteinuria and reduced renal function (Barutta et al., 2022; D'Agati et al., 2011; Sangameswaran et al., 2023; Sun et al., 2021). In idiopathic FSGS, which is the most common type, inflammation and fibrogenesis are thought to play important roles after podocyte injury since infiltration of inflammatory cells and activation of fibroblasts stimulate the production and deposition of extracellular matrix (ECM) components, including type I and type III collagen (Liu, 2011). Over time, these processes lead to tubulointerstitial fibrosis and, ultimately, end‐stage renal disease (ESRD) (Liu, 2011).

In rat models of FSGS, single or repeated injections of puromycin aminonucleoside (PAN) induce acute and chronic changes in glomerular morphology that resemble human nephrotic syndrome (Diamond & Karnovsky, 1986). Within 1 to 2 weeks after a single PAN treatment, histologic changes are evident, including proliferation of mesangial cells with matrix expansion, destruction of glomerular capillary lumen, development of adhesions between the glomerular tuft and Bowman's capsule, and swelling and bleb formation in glomerular epithelial cells. After several months, glomeruli show segmental areas of mesangial proliferation or glomerulosclerosis/hyalinosis in outer cortical and juxtamedullary areas, respectively. In addition, split renal function studies in rats with unilateral kidney perfusion with PAN have demonstrated that treated kidneys develop proteinuria and significantly reduced glomerular filtration rate and renal plasma flow in contrast to untreated kidneys in the same animal (Chandra et al., 1984). These histologic and functional changes may be therapeutically reversible. For example, darbepoetin, an erythropoietin analog, reverses podocyte injury (e.g., foot process retraction and effacement) and reduces proteinuria in PAN‐treated rats (Eto et al., 2007).

For patients with primary FSGS, first‐line treatment is high‐dose oral glucocorticoids (e.g., prednisolone) or other immunosuppressants, such as calcineurin inhibitors (Kidney Disease: Improving Global Outcomes Glomerular Diseases Work Group, 2021; Sangameswaran et al., 2023). Adrenocorticotropic hormone (ACTH) is an alternative treatment for patients who cannot tolerate or do not respond to these treatments (Kidney Disease: Improving Global Outcomes Glomerular Diseases Work Group, 2021). Acthar® Gel (repository corticotropin injection), which is a mixture of ACTH analogs (primarily N‐25 deamidated ACTH) and other peptides derived from porcine pituitary extract (Mallinckrodt Pharmaceuticals, 2021), may improve proteinuria and renal damage due to FSGS as shown in a rat PAN model of FSGS and patients with FSGS treated with Acthar. Previous studies have shown that the effects of Acthar are distinct from ACTH_1‐24_ and glucocorticoids, and suggest that Acthar has an immunomodulatory mechanism rather than a steroidogenic mechanism (Poola et al., 2022; Wang et al., 2021; Wright & Hayes, 2023). To further elucidate the differences between Acthar and glucocorticoids, we performed microarray analysis to identify changes in gene expression in the kidney cortex following treatment with Acthar or methylprednisolone (MP) versus control in a rat PAN model of FSGS.

## 2 MATERIALS AND METHODS

All study procedures, including for animal use welfare, were performed in accordance with the National Institutes of Health's Guide for the Care and Use of Laboratory Animals. The study protocol was reviewed and approved by the Institutional Animal Care and Use Committee of Mallinckrodt Pharmaceuticals.

Rats were housed in standard shoebox cages (16 × 8 × 8 inches; 3 or 4 animals each) with ambient temperatures between 65°F and 85°F. All study animals were kept on a 12‐h light/dark cycle, had ad libitum access to rat chow pellets (LabDiet 5001) and water, and were provided with enrichment devices. Animals were observed daily to assess general health and well‐being and were weighed at least twice per week.

The in‐life portion of the study lasted 8 weeks using 6‐to‐8‐week‐old female Sprague–Dawley rats (Envigo RMS; Research Resource Identifier [RRID]: IMSR_CRL:400) weighing 165–210 g on Day 0. All animals, except the PAN‐naive control group, received intravenous puromycin aminonucleoside (Cayman Chemical #15509) 50 mg/kg on Day 0, followed by a 20 mg/kg booster on Days 14, 21, and 28. Study treatment was initiated on Day 7 and continued until Day 56 in 3 cohorts: (Fogo, 2015) Acthar Gel (Mallinckrodt Pharmaceuticals) 60 IU/kg (5 mL/kg; *n* = 5), administered subcutaneously every 48 h, (Sangameswaran et al., 2023) MP 2 mg/kg (MP Biomedicals #151671), administered orally every 24 h (*n* = 5), or (Barutta et al., 2022) saline 5 mL/kg (*n* = 4), administered subcutaneously every 48 h. Blood pressure was assessed using a tail cuff before the start of the study and at 14‐day intervals during the study.

On Day 56, animals were anesthetized with isoflurane and sacrificed by cardiac exsanguination, and kidneys were removed. Kidney cortex tissue was minced into ~1–2 mm pieces, and approximately 10–20 mg of tissue was solubilized in RNAlater (Thermo Fisher #AM7020). Samples were flash frozen in liquid nitrogen and stored at −80°C. Frozen tissue samples were not weighed to minimize tissue breakdown; gene expression was normalized by equal concentrations of purified RNA from each sample on the gene expression array. For RNA extraction, tissues were thawed on wet ice and transferred into Trizol solution (Invitrogen #15596026) for homogenization using the Omni Bead Ruptor Elite (OMNI International, #19‐040E) with 1.4 mm ceramic beads (Omni International, #19–645), and then subjected to phenol/chloroform extraction. The aqueous phase was removed and added to Qiagen RNeasy 96 QIAcube HT extraction kit columns (Qiagen #7471) on a QIAcube HT automated liquid handling platform (Qiagen #9001896; RRID:SCR_020419) according to the manufacturer's protocol. The purity and concentration of extracted RNA were assessed by the ratio of absorbance at 260 versus 280 nm using a Nanodrop 2000c spectrophotometer (Thermo Fisher Scientific; RRID:SCR_020309), and RNA integrity was evaluated by the ratio of 28S to 18S ribosomal RNA with an Agilent Bioanalyzer 2100 capillary electrophoresis instrument (RRID:SCR_018043).

Microarray analysis was performed by Genemarkers, LLC, using the Affymetrix Clariom™ S Assay for rats. Gene expression data were analyzed with Transcriptome Analysis Console software (TAC; version 4.0.2; Thermo Fisher Scientific; RRID:SCR_016519) using the default settings. Pathway analysis was performed using WikiPathways data available in TAC as of 10 December 2024. (Agrawal et al., 2024) Differences in gene expression were assessed for statistical significance (*p* < 0.05) using *F* tests in TAC.

## 3 RESULTS

**FIGURE 1:**
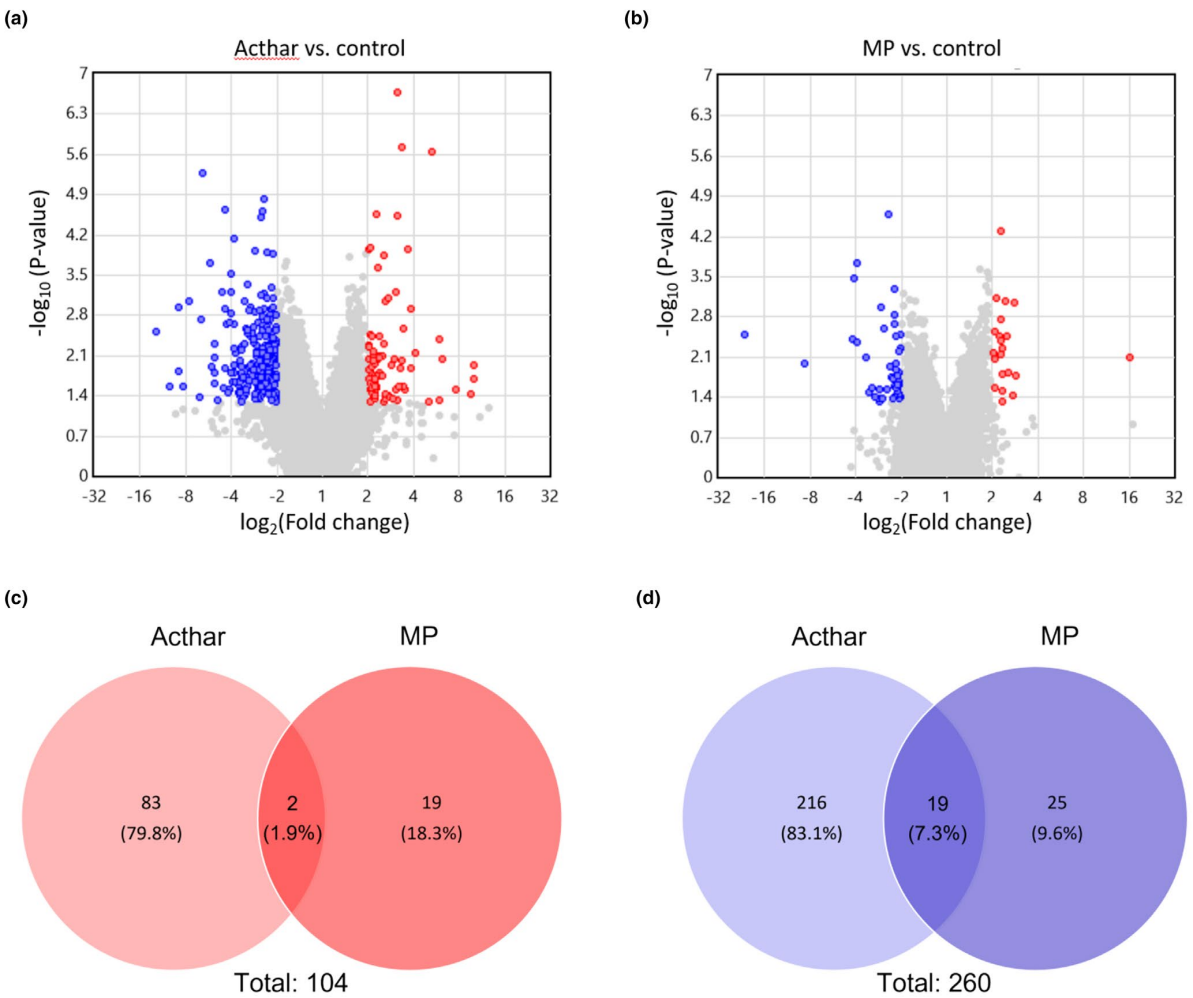
Effect of Acthar versus MP on gene expression in rat kidney cortex after 8 weeks of treatment. Volcano plots of significant ≥2‐fold changes in gene expression in rat kidney cortex following 8 weeks of treatment with Acthar (a) or MP (b) versus control. Venn diagrams of the number of unique and shared genes between Acthar and MP with significant ≥2‐fold increases (c) or decreases (d) in expression. Red color represents statistically significant ≥2‐fold increases in gene expression compared with the control. Blue color represents statistically significant ≥2‐fold decreases in gene expression compared with the control. Gray color represents <2‐fold increases or decreases in gene expression compared with the control. Statistical significance was *p* < 0.05 for Acthar or MP versus control. MP, methylprednisolone.

Microarray analysis of kidney cortex tissue revealed that Acthar significantly altered the expression (≥2‐fold) of more genes than MP (320 vs. 65; Figure 1a,b) compared with the PAN‐naive control. After Acthar treatment, 235 genes had significantly ≥2‐fold lower expression versus control, and 85 genes had significantly ≥2‐fold higher expression. After MP treatment, 44 genes had significantly ≥2‐fold lower expression versus control, and 21 genes had significantly ≥2‐fold higher expression. Acthar had 299 unique genes that were significantly ≥2‐fold upregulated or downregulated, compared with 44 genes with MP (Figure 1c,d). Only 21 genes were significantly ≥2‐fold upregulated or downregulated by both Acthar and MP.

Acthar significantly decreased the expression (≥2‐fold) of more genes related to the immune system, inflammation, and fibrosis than MP (74 vs. 9, respectively; Table 1). Only seven of these genes were significantly downregulated by both Acthar and MP.

**TABLE 1:** Genes associated with immune response, inflammation, and tissue remodeling with significantly lower expression (≥2‐fold) after treatment with Acthar or MP versus control.

General function	Acthar 60 IU/kg	MP 2 mg/kg
Immune/inflammation	*Alox5ap, C1qb, C1qc, C1r, C1s, C3, C4a, C4b, C7, Ccl6, Ccl21, Ccr2, Cd180, Cd37, Cd4, Cd53, Cd68, Cfh, Cfi, Cfp, Cxcl13, Cxcr3, Cybb, Fcgr2b, Hspb1, Icam1, Ifi27l2b, Il1rl1, Il6r, Il7r, Il10ra, Itk, Lcp2, Lsp1, Lyn, Map3k1, Mpeg1, Ncf1, Nfam1, Nfkbia, Prkcb, Ptprc, Rac2, Socs3, Tcrb, Tlr13, Xcr1*	*Cd4, Cxcl13, Cxcr3, Fcrl2, Il1b, Xcr1*
Tissue remodeling	*Actn1, Cd47, Cdkn1a, Clu, Col1a2, Col6a3, Col8a1, Fbn1, Fgb, Fgl2, Fmod, Itga11, Jun, Lamc2, Mfap4, Mgp, Mmp12, Mmp14, Pcolce, Pdgfra, Rac2, Rbl1, Serpine1, Serpinh1, Sox9, Spp1, Vim*	*Fgl2, Fmod, Mmp12*

*Note*: All genes listed demonstrated significant downregulation compared with the control (*p* < 0.05).

Abbreviation: MP, methylprednisolone.

**FIGURE 2:**
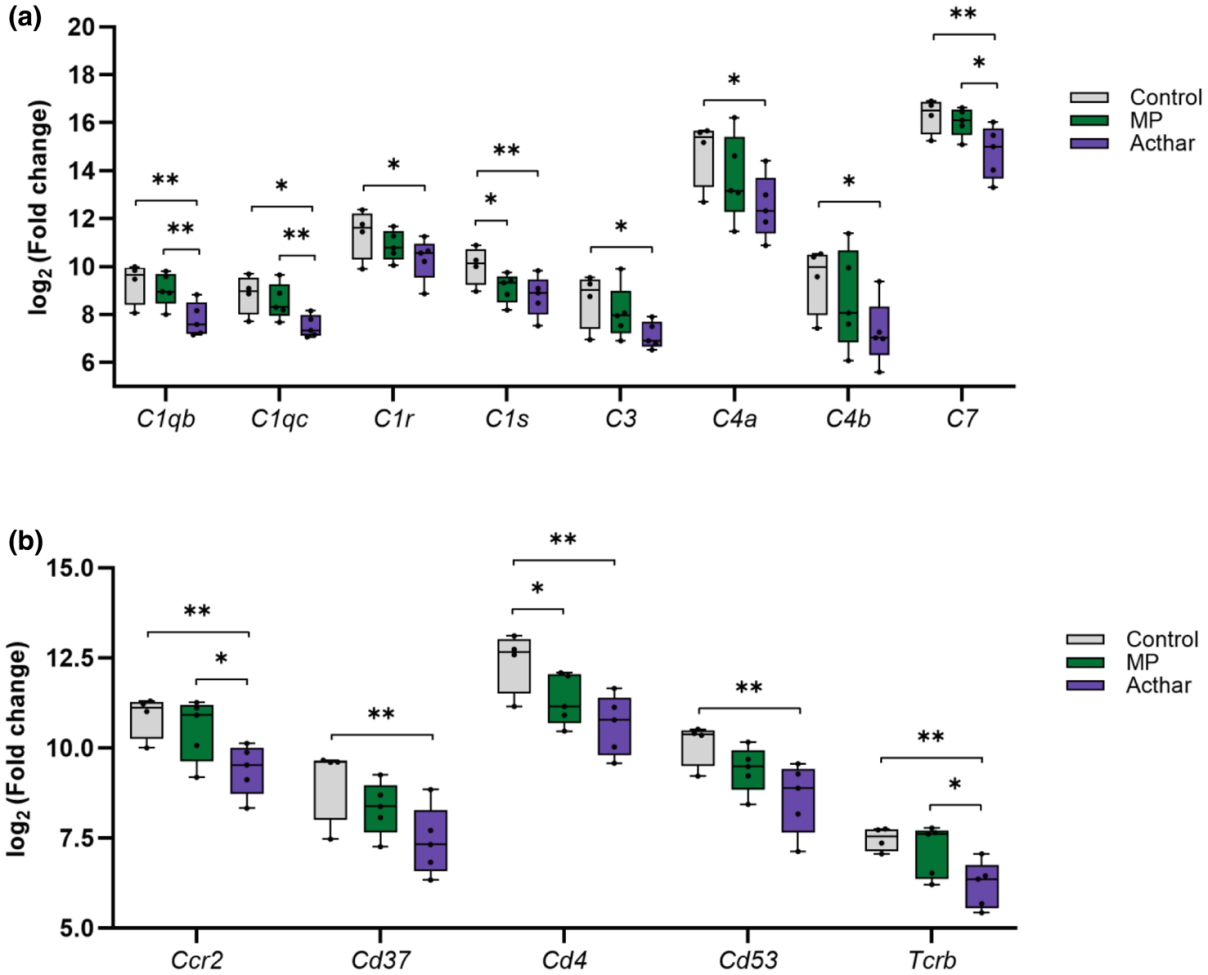
Change in the expression of genes related to the complement system (a) or immune system (b) after treatment with Acthar or MP versus control. *p < 0.05, **p < 0.01. MP, methylprednisolone.

Acthar showed greater decreases than MP in the expression of several complement and immune‐related genes relative to control (Figure 2). In addition, Acthar resulted in significantly larger fold changes in expression than MP of *C1qb* and *C1qc* complement genes and *Ccr2* and *Tcrb* immune‐related genes (Figure 2).

**FIGURE 3:**
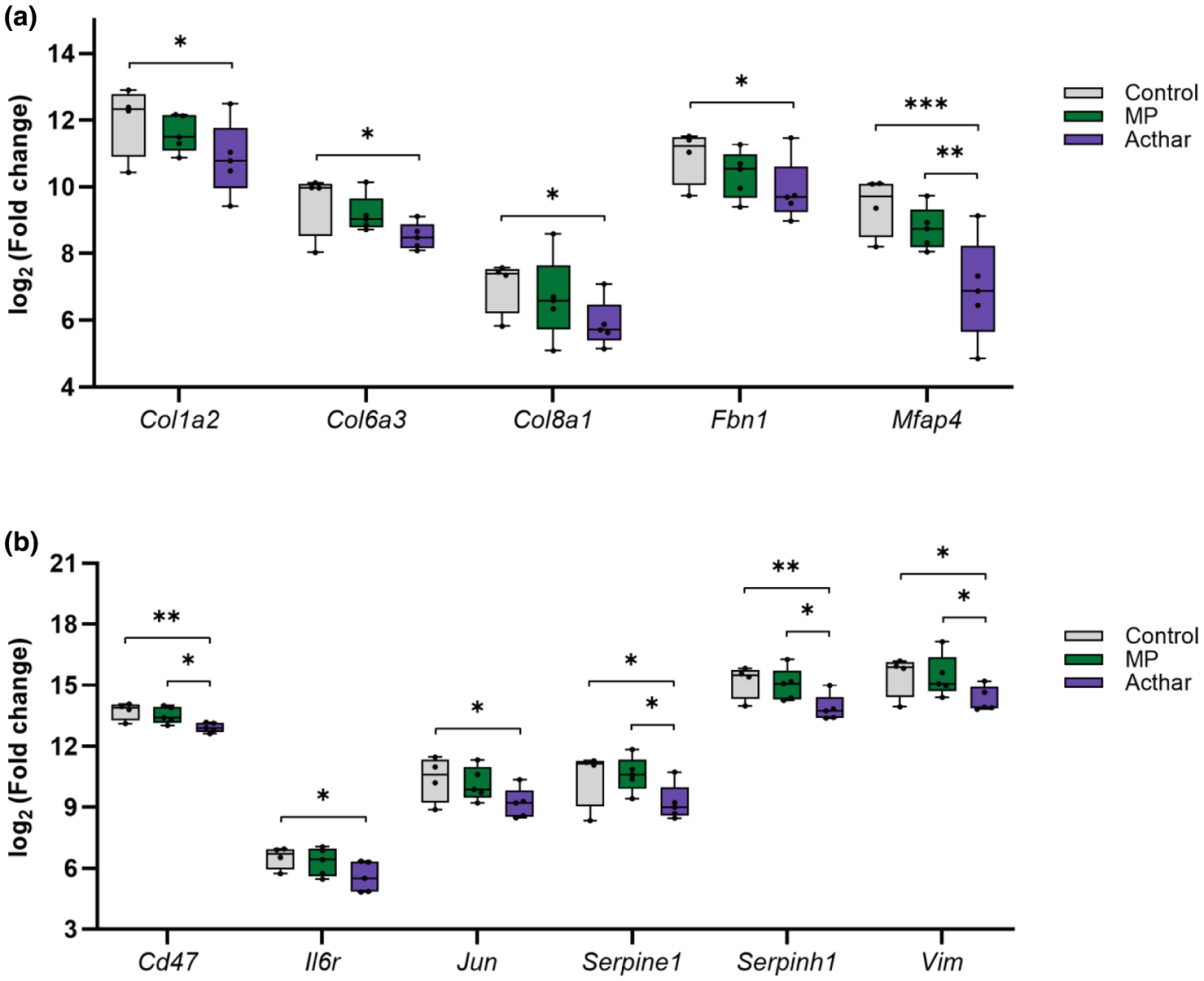
Change in the expression of genes related to fibrosis and ECM (a) or fibroblasts (b) after treatment with Acthar or MP versus control. *p < 0.05, **p < 0.01; ***p < 0.001. ECM, extracellular matrix; MP, methylprednisolone.

Acthar also demonstrated greater decreases than MP in the expression of several genes related to fibrosis, ECM, and fibroblasts relative to control (Figure 3). Among the fibrosis and ECM genes, Acthar resulted in a significantly larger fold change in gene expression than MP of *Mfap4*, which encodes a fibrotic ECM protein. Among fibroblast genes, Acthar resulted in significantly larger fold changes in gene expression than MP of *Cd47*, *Serpine1*, *Serpinh1*, and *Vim* (Figure 3).

Pathway analysis indicated that genes that are differentially expressed after treatment with Acthar versus control are significantly associated with 21 biological pathways (Table S1). Approximately half (*n* = 10; 47.6%) of these pathways are immune‐related, including complement and coagulation cascades (*p* < 0.0001), complement activation (classical pathway) (*p* < 0.0001), and T‐cell receptor signaling pathway (*p* = 0.0001). In contrast, differentially expressed genes after treatment with MP versus control were significantly associated with six biological pathways, of which only two (cytokines and inflammatory response [*p* = 0.0028] and spinal cord injury [*p* = 0.0359]) were immune‐related (Table S2).

## 4 DISCUSSION

Our results show that Acthar significantly changed the expression of substantially more genes than MP did relative to control. Of note, Acthar significantly downregulated the expression of genes associated with the immune system, inflammation, and fibrosis. For some of these genes, the fold change in expression was significantly greater for Acthar than for MP. Very few genes were significantly regulated by both Acthar and MP, highlighting the differentiation of the two drugs, which is an important finding due to the common misconception that Acthar functions in the same manner as glucocorticoids. These findings suggest that Acthar functions via immunomodulation rather than exclusively through steroidogenesis.

This hypothesis is supported by several lines of evidence in humans and animal models. In healthy subjects treated with Acthar, cortisol levels were only slightly elevated beyond normal endogenous levels (Poola et al., 2022; Wang et al., 2021). In vitro, Acthar inhibits B‐cell proliferation and immunoglobulin production and reduces the production of inflammatory cytokines (e.g., tumor necrosis factor α [TNFα], interleukin‐6 [IL‐6]) in macrophages (Healy et al., 2017; Olsen et al., 2015). In a murine model of T‐cell activation, Acthar reduced the activation of CD4^+^ and CD8^+^ T cells as well as the production of cytokines (interleukin‐2, interferon γ, and TNFα), which are involved in inflammatory immune responses in autoimmune disorders, such as multiple sclerosis (Wright & Hayes, 2023).

In our study, Acthar decreased the expression of genes involved in inflammation and tissue remodeling, which contribute to the pathogenesis of FSGS. Compared with control, Acthar significantly decreased the expression of eight complement genes, including four genes (*C1qb*, *C1qc*, *C1r*, and *C1s*) that encode components of the C1 complex, which initiates the classical complement pathway, and three genes (*C3, C4a*, and *C4b*) that encode downstream proteins (Noris & Remuzzi, 2013). Another gene with reduced expression, *C7*, encodes a component of the terminal membrane attack complex that promotes inflammation and cell lysis (Noris & Remuzzi, 2013). Furthermore, Acthar resulted in a significantly greater fold reduction in the expression of *C1qb* and *C1qc* than MP, which suggests that Acthar may inhibit the initiation of the complement cascade more strongly than MP. These results are consistent with previous preclinical and clinical studies of FSGS that suggest that the classical complement pathway is overactivated in glomerulosclerosis and support the use of Acthar to slow FSGS progression (Huang et al., 2020; Thurman et al., 2015; Turnberg et al., 2006; van de Lest et al., 2019; Zhang et al., 2016).

Acthar also decreased the expression of genes related to tissue remodeling. These genes encode components of collagen (*Col1a2*, *Col6a3*, and *Col8a1*) and other ECM proteins (*Fbn1* and *Mfap4*), a molecular chaperone involved in collagen formation (*Serpine1*), and proteins involved in the accumulation of ECM (*Serpinh1* and *Il6r*) (Cui et al., 2020; Ghosh & Vaughan, 2012; Ito & Nagata, 2017; Li et al., 2021; Pan et al., 2020). Vimentin, which is encoded by another gene downregulated by Acthar, *Vim*, is an intracellular cytoskeletal protein in mesenchymal cells, including fibroblasts, which are the primary cellular component and source of ECM in fibrotic human kidney tissue (Bucki et al., 2023; Kuppe et al., 2021). In addition, vimentin is a marker of the epithelial–mesenchymal transition, which is one of several mechanisms that generate myofibroblasts in the kidney and is associated with podocyte dysfunction leading to glomerulosclerosis (LeBleu et al., 2013; Liu, 2010). Moreover, the ability of Acthar to reduce the expression of *Mfap4*, *Serpine1*, *Serpinh1*, and *Vim* significantly more than MP suggests that Acthar may inhibit inflammation and fibrosis development more than MP. This hypothesis is further supported by the decreased expression of *Jun* and *Cd47* in response to Acthar treatment. Activation of Jun, a transcription factor in the JNK pathway, promotes transcription of genes involved in many fibrotic diseases, including renal disease, and is associated with increased expression of CD47, an anti‐phagocytic protein that promotes fibrosis (Cui et al., 2020; Grynberg et al., 2017; Wernig et al., 2017). Because renal fibrosis correlates strongly with reduced renal function and is a predictor of faster progression to dialysis in ESRD (Martínez‐Klimova et al., 2019; Menn‐Josephy et al., 2016), treatment with Acthar may be more effective than MP in delaying progression from FSGS to ESRD.

In addition to decreasing the expression of genes involved in inflammation and tissue remodeling, Acthar significantly reduced the expression of several genes with broad immune functions compared with the control. These genes included *Ccr2, Cd37*, and *Cd53*, which encode proteins involved in the regulation of immune cell activation, proliferation, and signaling (Dunlock, 2020; Singh et al., 2021). Acthar also decreased the expression of *Tcrb* and *Cd4*, which encode the T‐cell receptor (β subunit) and its co‐receptor CD4 that recognize antigens presented by major histocompatibility complex class II molecules (Murphy & Weaver, 2017). In mice with unilateral ureteric obstruction, a model of renal interstitial fibrosis, CD4 expression is associated with increased gene expression and deposition of type I collagen in kidney tissue (Tapmeier et al., 2010). The observation that Acthar reduces the expression of *Ccr2* and *Tcrb* significantly more than MP suggests that Acthar may suppress the immune response to a greater degree than MP.

The differences in the effects of Acthar versus MP on gene expression were in agreement with the pathway analyses, which suggested that Acthar may modulate immune‐related pathways to a greater extent than MP, although there is some overlap. Our findings are also generally consistent with a recent transcriptome analysis of kidney biopsy tissue from patients with diabetic nephropathy, which showed enrichment of pathways involved in inflammation, complement activation, and ECM organization (Levin et al., 2020). In addition, many human orthologs of the rat genes that had more significant decreases in expression after Acthar treatment compared with MP, including *C1qb*, *C1qc*, *Ccr2*, *Mfap4*, and *Vim*, were upregulated in the transcriptome analysis. This agreement suggests that the differential effects of Acthar versus MP in the rat PAN model of FSGS can be translated to patients with FSGS.

It should be noted that this was a preliminary study and additional experiments are needed to confirm the results. In particular, protein expression analyses (e.g., Western blot) are needed to assess whether the observed changes in gene expression translate to changes in protein expression.

Collectively, the gene expression findings support the hypothesis that Acthar is an immunomodulator with a distinct mechanism of action from that of glucocorticoids such as MP. In addition, the differential effects in the renal cortex of a rat model of FSGS suggest that Acthar may be more effective than MP in reducing inflammation and fibrosis in FSGS, which could slow disease progression. Finally, the consistency between gene expression analyses in this rat model and patients with renal disease supports continued study of Acthar as a treatment for patients with FSGS.

## AUTHOR CONTRIBUTIONS

K.H. and D.W. conceived and designed the research; K.H. and Genemarkers performed the experiments; K.H., D.W., Genemarkers, and MedLogix Communications analyzed the data; K.H. and D.W. interpreted the results of the experiments; MedLogix Communications developed the manuscript (main text and figures); K.H. and D.W. reviewed the manuscript; K.H. and D.W. approved the final version of the manuscript.

## FUNDING INFORMATION

Kyle Hayes is an employee of Mallinckrodt Pharmaceuticals. Dale Wright is a former employee of Mallinckrodt Pharmaceuticals.

## ETHICS STATEMENT

All study procedures, including for animal use welfare, were performed in accordance with the National Institutes of Health's Guide for the Care and Use of Laboratory Animals. The study protocol was reviewed and approved by the Institutional Animal Care and Use Committee of Mallinckrodt Pharmaceuticals. There are no human participants in this article and informed consent is not required.

## Supporting information


**Table S1–S2:** Supporting Information.

## Data Availability

The datasets from this study are available from the corresponding author upon reasonable request.
