# Supplementary material for: Microarray analysis of the effects of Acthar Gel versus methylprednisolone in a model of focal segmental glomerulosclerosis in female rats
Source: Physiol Rep. 2025 Apr 13;13(7):e70321. doi: 10.14814/phy2.70321 (PMC11994893; doi:10.14814/phy2.70321)
Supplement: Supplementary file 1 — Table S1–S2: Supporting Information. [file PHY2-13-e70321-s001.docx]

**Supplemental Table 1**. Biological pathways and associated genes significantly upregulated (≥2-fold) or downregulated (≤2-fold) after treatment with Acthar vs control

| **Pathway^a^** | **Genes upregulated** | **Genes downregulated** | **Significance** | **P value** |
| --- | --- | --- | --- | --- |
| Complement and coagulation cascades | — | *Cfi, C7, Serpine1, C1qb, Kng1, C3, Cfh, C4a, Fgb, C1r, C1s* | 8.92 | <0.0001 |
| Complement activation - classical pathway | — | *C1s, C1qb, C1r, C1qc, C3, C7* | 7.18 | <0.0001 |
| T-cell receptor signaling pathway | — | *Map3k1, Cd4, Lyn, Rac2, Nfam1, Lcp2, Itk, Ptprc, Jun* | 3.92 | 0.0001 |
| Spinal cord injury | — | *Cd47, Btg2, Mmp12, Ccr2, Icam1, Sox9, Vim, Gja1* | 3.90 | 0.0001 |
| IL-5 signaling pathway | — | *Prkcb, Nfkbia, Lyn, Jun, Alox5ap, Icam1* | 3.32 | 0.0005 |
| Calcium regulation in cardiac cells | *Casq1, Gjb5* | *Prkcb, Rgs1, Gnb4, Gja1, Kcnj3, Cacnb3* | 2.93 | 0.0012 |
| B-cell receptor signaling pathway | — | *Lyn, Itk, Lcp2, Fcgr2b, Jun, Nfkbia, Prkcb, Ptprc* | 2.69 | 0.0020 |
| Eicosanoid synthesis | — | *Alox5ap, Alox15b, Ptgs2* | 2.60 | 0.0025 |
| Myometrial relaxation and contraction pathways | — | *Jun, Rgs1, Prkcb, Cnn2, Gnb4, Gja1, Cacnb3* | 2.21 | 0.0062 |
| Focal adhesion | — | *Actn1, Col1a2, Lamc2, Spp1, Pdgfra, Rac2, Jun, Itga11* | 2.14 | 0.0072 |
| Endochondral ossification | — | *Spp1, Serpinh1, Sox9, Mgp* | 1.92 | 0.0121 |
| Type II interferon signaling (IFNγ) | — | *Socs3, Cybb, Icam1* | 1.88 | 0.0132 |
| IL-3 signaling pathway | — | *Lyn, Prkcb, Socs3, Rac2, Hspb1* | 1.79 | 0.0163 |
| Fas pathway and stress induction of HSP regulation | — | *Hspb1, Jun, Map3k1* | 1.78 | 0.0166 |
| IL-2 signaling pathway | — | *Lyn, Cd53, Icam1, Socs3* | 1.62 | 0.0241 |
| NFE2L2 signaling pathway | *Gstm2, Gclc, Gsta3, Gstm1* | *Cdkn1a, Prkcb, Jun, Map3k1* | 1.47 | 0.0339 |
| Blood clotting cascade | — | *Serpine1, Fgb* | 1.47 | 0.0339 |
| Glutathione metabolism | *Gclc, Gstm2* |  | 1.43 | 0.0372 |
| TGFβ signaling pathway | — | *Spp1, Serpine1, Jun* | 1.42 | 0.0384 |
| Adipogenesis | — | *Rbl1, Serpine1, Cdkn1a, Socs3, Klf6* | 1.38 | 0.0422 |
| G protein signaling pathways | *Pde4c* | *Prkcb, Gna14, Kcnj3* | 1.35 | 0.0444 |

^a^Pathways were identified using WikiPathways data as of December 10, 2024.

HSP indicates heat shock protein; IFN, interferon; IL, interleukin; NFE2L2, nuclear factor erythroid-derived 2‑like 2; TGFβ, tumor growth factor beta.

**Supplemental Table 2**. Biological pathways and associated genes significantly upregulated (≥2-fold) or downregulated (≤2-fold) after treatment with MP vs control

| **Pathway^a^** | **Genes upregulated** | **Genes downregulated** | **Significance** | **P value** |
| --- | --- | --- | --- | --- |
| Cytokines and inflammatory response | — | *Il1b, Cd4* | 2.55 | 0.0028 |
| Metapathway biotransformation | *GSTA1, Cyp24a1, Gstm5* | *Cyp1b1* | 2.08 | 0.0083 |
| G protein signaling pathways | — | *Kcnj3, Akap12* | 1.54 | 0.0291 |
| Spinal cord injury | — | *Il1b, Mmp12* | 1.45 | 0.0359 |
| Estrogen metabolism | *GSTA1* | *Cyp1b1* | 1.40 | 0.0401 |
| Burn wound healing | *Car4* | *Il1b* | 1.34 | 0.0453 |

^a^Pathways were identified using WikiPathways data as of December 10, 2024.
